# In the June 2015 issue

**DOI:** 10.1590/1980-57642015DN92000001

**Published:** 2015

**Authors:** Ricardo Nitrini

**Affiliations:** Editor-in-Chief

In this issue we are publishing two reviews, nine original papers, one history note and two case reports.

**Jacinto et al.** evaluated dementia teaching in undergraduate courses of Medicine in Brazil and abroad. Teaching on dementia has been included in undergraduate programs of Medicine in developed countries for at least a decade, while specific data on the subject are lacking in most countries. In Brazil, Geriatrics is not part of the curriculum in many medical schools, which led the authors to infer that teaching on dementia is very limited.

**Matioli and Nitrini** reviewed the connection of diabetes mellitus and/or brain insulin resistance/deficiency with Alzheimer's disease. Several mechanisms, including impairment of glucose transport, oxidative and endoplasmic reticulum stress, mitochondrial dysfunction, accumulation of advanced glycation end products with increased production of neuro-inflammation and activation of pro-apoptosis cascade may contribute to neurodegeneration. These are important issues for planning new strategies to prevent and treat AD in the future.

**Ferretti-Rebustini et al.** investigated which demographic and clinical factors were associated with morphometric brain measurements in a brain bank. Head circumference was independently associated with low brain weight and volume. The authors found that female gender, age and arterial hypertension were associated with low adjusted brain volume whereas years of schooling may have a protective effect.

**Brigola et al.** performed a systematic review of the literature to analyze the relationship between cognition and frailty in the elderly. All 19 studies selected showed that components of frailty and the cognitive domains were correlated. Cognitive impairment was found to be more frequent in the frail elderly, and the greater the frailty, the higher the risk of cognitive impairment and dementia.

**Zimmermann et al.** evaluated the performance of adults on three tests of executive function (Modified Wisconsin Card Sorting, Stroop color and word and Digit Span test). Participants were divided into young, middle-aged, and older adults and into two education levels (5 to 8 years and 9 or more years of schooling). Education had a much greater impact on performance in these tests than aging, reinforcing the need for norms stratified by education to assess executive functions.

**Mendes-Santos et al.** analyzed the performance of 100 cognitively normal elderly on the Clock Drawing Test to evaluate inter-rater reliability. The scores followed an algorithm method adapted from that described by Sunderland et al. (1989). Inter-rater reliability was high, but authors suggested that more nuanced evaluation criteria should be developed. They also proposed the incorporation of an evaluation able to disclose differences in levels of impairment due to visuoconstructive or executive ability decrease during aging.

**Bolognani et al.** presented three alternative short stories for the logical memory test from the Wechsler Memory Scale to minimize learning effects when repeated testing is required for longitudinal evaluations. The alternative stories were developed following the same structure as the originals, and corresponded well thematically and structurally to the Brazilian versions of the original stories. These new versions are useful for longitudinal evaluations.

**Assis Faria et al.** performed a systematic review of the literature to find the most frequently used tests for assessing executive functions in the elderly over the last five years. The authors found seven commonly used tests assessing mental flexibility, verbal fluency, planning, working memory, and inhibitory control. These results may aid future research and help build evaluation protocols for executive functions, taking into account different levels of education.

**Cardoso et al.** presented the methods used to develop the Brazilian adaptation of the Hotel Task, an ecological instrument for assessing executive functions. The adaptation followed strict criteria, from translation to pilot studies, and even included evaluation by an expert in hotel management. Although further research is needed to investigate its reliability, validity and accuracy, these preliminary results are important.

**Ferreira et al.** investigated cognitive impairment after first stroke in 45 patients 6-10 months prior to evaluation. Cognitive impairment was frequent and found to be associated with female gender, age and stroke severity. However, the side of hemispheric stroke was not associated with frequency of cognitive impairment in this sample.

**Dutra et al.** applied the Brazilian version of the Functional Activities Questionnaire of Pfeffer et al. to verify the reliability and accuracy of the instrument. The questionnaire showed good accuracy compared to an activities of daily living scale and a cognitive assessment test. The inter-rater and intra-rater reliability were also high, showing that this instrument, which is very simple to apply, is useful for the evaluation of the elderly in Brazil.

**Engelhardt and Grinberg** highlighted the importance of Alois Alzheimer's work on vascular brain diseases, particularly vascular dementia. According to the authors, Alzheimer described the differences between general paresis due to neurosyphilis and arteriosclerotic dementia and also recognized that senile dementia was a highly heterogeneous condition which had vascular and neurodegenerative subtypes.

**Angst et al.** reported a case of autoimmune limbic encephalitis associated with systemic lupus erythematous, a rare association. In this case, there was also a past medical history of cancer (endometrial adenocarcinoma and papillary urothelial carcinoma). However, the marked improvement in encephalitis with immunotherapy, not seen with paraneoplastic limbic encephalitis, essentially ruled out this alternative diagnosis. The presence of antibodies against neuronal membrane was a reasonable hypothesis for the observed improvement.

**Camargo et al.** described the evolution of a patient with Alzheimer's disease while using an anti-TNF-α agent for rheumatoid arthritis. Although the patient was also treated with donepezil and was engaged in a non-pharmacological treatment intervention, the significant cognitive improvement during the follow-up period led the authors to raise the hypothesis of a synergistic effect of anti-TNF-α with the other treatments used.

**Ricardo Nitrini** Editor-in-Chief

